# Invoking and identifying task-oriented interlocutor confusion in human-robot interaction

**DOI:** 10.3389/frobt.2023.1244381

**Published:** 2023-11-20

**Authors:** Na Li, Robert Ross

**Affiliations:** School of Computer Science, Technological University, Dublin, Ireland

**Keywords:** confusion detection, multimodal modeling, user engagement, situated dialogue, wizard-of-oz, social robot

## Abstract

Successful conversational interaction with a social robot requires not only an assessment of a user’s contribution to an interaction, but also awareness of their emotional and attitudinal states as the interaction unfolds. To this end, our research aims to systematically trigger, but then interpret human behaviors to track different states of potential user confusion in interaction so that systems can be primed to adjust their policies in light of users entering confusion states. In this paper, we present a detailed human-robot interaction study to prompt, investigate, and eventually detect confusion states in users. The study itself employs a Wizard-of-Oz (WoZ) style design with a Pepper robot to prompt confusion states for task-oriented dialogues in a well-defined manner. The data collected from 81 participants includes audio and visual data, from both the robot’s perspective and the environment, as well as participant survey data. From these data, we evaluated the correlations of induced confusion conditions with multimodal data, including eye gaze estimation, head pose estimation, facial emotion detection, silence duration time, and user speech analysis—including emotion and pitch analysis. Analysis shows significant differences of participants’ behaviors in states of confusion based on these signals, as well as a strong correlation between confusion conditions and participants own self-reported confusion scores. The paper establishes strong correlations between confusion levels and these observable features, and lays the ground or a more complete social and affect oriented strategy for task-oriented human-robot interaction. The contributions of this paper include the methodology applied, dataset, and our systematic analysis.

## 1 Introduction

Socially interactive robotic agents have been applied to assisting people in many different areas in the last 10 years, *e.g.*, language learning (Saeki et al., 2022), tour guides (Duchetto et al., 2019), helping children with autism therapy (Zhanatkyzy et al., 2023a), to name but a few. In such social human-robot interaction (HRI) scenarios, the autonomous social robot must assess and maintain the engagement of the interlocutor in terms of determining the context of interaction and inference of the meaning of any social interactive signals. Moreover, in the ideal case, not only should the robotic system observe and model the user, but it should be able to alter its own actions in response to the user affect state, and predict the effects of its own behaviors (Lugrin et al., 2022, chapter 16.3).

Many different branches of computational linguistics and human-computer interaction (HCI) have been applied in the pursuit of effective human-robot interaction (HRI). Early work focused invariably on the analysis of the structure of language itself, and the various levels at which there were interactions between language, the task competence and actions of the robotic agent, and its perception of the environment including the user. Such work, which is based on disciplines such as conversational analysis and dialogue modeling, often straddled a boundary between linguistics, epistemology, and engineering (Lugrin et al., 2022, chapter 15).

In recent times, the HCI and HRI communities have also explored the richness of audio and visual data to estimate emotion and other cues of communicative function. This work recognizes that social communication is mediated not only by pure ideational language, but by a range of modalities and communicative functions, including facial expression, body language, and emotional intonation in speech. In much of this work, there is an assumption that designing an engaged conversational HRI is critical. However, while some studies have examined emotion estimation in HRI that can promote user engagement (Busso et al., 2008; Celiktutan et al., 2017); there is relatively little research to date on comprehensive user engagement estimation in either situated conversational HRI or spontaneous conversation, *e.g.*, Ben Youssef et al. (2017), and Benlamine and Frasson (2021).

While emotion estimation and engagement studies in general are essential for effective HRI, our work is focused on a particular aspect of the mechanisms for successful engagement and task completion in practical HRI tasks. In particular, we are focused on the notion of confusion, and more specifically, what it means for a user to be confused in a joint task and how that confusion state can be both modeled and mitigated. Confusion has relationships with, but does not always correlate with, more traditional transient mental states such as engagement or emotion. For example, a user can be engaged with both a task and confused, but often confusion will in time lead to a reduction in engagement and ultimately satisfaction. Turning to the general concept of emotion, confusion in its worst case might be associated with anger, but in fact confusion may also correlate with a neutral emotion or even despondency—depending on the relationship between the confused, the interlocutor and the nature of the task at hand (D’Mello et al., 2014). Given these qualities, to date, there have been a number of models proposed that attempt to structure the modes of confusion. For example, some researchers in learning have suggested that confusion can be a phenomenon with multiple levels, or even with four classes from very high to very low (Arguel and Lane, 2015; Samani and Goyal, 2021). Taking a similar approach, Lodge et al. (2018) proposed two states of confusion, *i.e.*, productive confusion and unproductive confusion in learning.

Due to its role in accounting for disengagement and even task success, the identification of confusion and its mitigation have value both in the HCI and HRI communities. While some work has been conducted on the modeling of confusion, as just discussed, and on the *in-situ* study of confusion, much of that work has typically been focused on pedagogy or androgogy, *e.g.*, language learning (Saeki et al., 2022). Specific studies in the area of confusion, *e.g.*, Grafsgaard et al. (2011), Zhou et al. (2019) and Kumar et al. (2019) have generally focused on online learning in specific environments such as AutoTutor, ITS (Intelligent Tutoring Systems), MOOCs (Massive Open Online Courses), or serious games.

To address this gap in empirical studies and the modeling of confusion in general, and specifically in the case of human-robot interaction, our research is focused on the systematic triggering of confusion in task-oriented interactions, observing and analyzing the user in such interactions, and building models to both detect user confusion states and feed these into policies to mitigate the confusion. Building on an earlier pilot study (Li and Ross, 2023), the work presented in this paper has been constructed to elicit different confusion states in a user participating in a collaborative task in order to determine if confusion is a simple binary state, or if it is in fact a multifaceted state associated with different levels of engagement and task completion rates. The hypothesis of this work is **user confusion does produce physical and behavioral manifestations and that these manifestations can be identified and learned**.

To be more concrete, in this paper we set out a model of confusion as a two-tier mental state (productive confusion and unproductive confusion), and built out a deeper HRI study to attempt to elicit and then detect these different levels of confusion. To approach this study, a Wizard-of-Oz (WoZ) experiment was designed, which improved upon some limitations of our pilot study, and subsequently analyzed multimodal data against self-reported states and task conditions. The contributions of this work are thus: 1) a multilevel framework for modeling confusion states in HRI; 2) a WoZ HRI study design for different states of confusion analysis; 3) an analysis of correlation between measured features and confusion states; and 4) our feature data and user speech transcripts that are made public to promote further research on confusion and mental state perception in the field of HRI^1^. We also briefly outline the approach we are taking to mitigate confusion through a structured interaction policy.

## 2 Related work

The study of the concept of confusion and being able to estimate a user’s confusion state during interactions straddles disciplines including pedagogy, linguistic pragmatics, HCI and HRI. The study and modeling of confusion itself also have overlaps with related topics such as emotion and engagement estimation. To explore the concepts and work which underpin our studies we will first delve into existing work related to confusion modeling and estimation, and then provide a brief overview of the state-of-the-art research in the related domains of emotion and engagement estimation.

### 2.1 Confusion definitions and detection

Unlike other mental concepts such as personality that have well-understood conceptualizations, specific models and definitions for confusion are more fluid—though with clear trends. For a simple definition-based understanding, confusion can simply mean “lack of clarity” (Gordon, 2006). However, as argued by D’Mello and Graesser (2014), the meaning of confusion can actually be much more complex, and has in different fields been described as a bonafide emotion, a knowledge emotion, an epistemic emotion, an affective state or a mere cognitive state. When confusion is defined as an epistemic emotion (Lodge et al., 2018), it is associated with blockages or impasses in a learning process. Gordon (2006) claimed that in education, confusion, or uncertainty, drives people to explore more, question previous beliefs, or even create a new perspective, because, as the authors pointed out, people generally will lack passion to clarify something that they already know and understand. Confusion as a state is also associated with the notion of cognitive disequilibrium. Yang et al. (2015) explained that cognitive disequilibrium is a state in which a user encounters obstacles in the normal flow of the learning process, making the user feel confused when encountering contradictory information that leads to uncertainties.

It should be noted that confusion can be equated with a form of uncertainty in educational contexts (Cumbal et al., 2020). Specifically, within the domains of social science and psychology, such uncertainty is related to the concept of “metaignorance state”, representing a state where individuals are conscious of their lack of knowledge (Smithson, 2012; Anderson et al., 2019). In such studies of metaignorance, there are said to be three sources of uncertainty: The first source is probability, caused by the inherent randomness or indeterminacy of future events; the second source is ambiguity, stemming from limitations in the reliability, credibility or sufficiency of available probability, linked to risk assessment and information quality; and the third source is complexity, resulting from intricate features within available information that are difficult to understand, *e.g.*, diverse potential causes or results (Han et al., 2011). As such it can be seen that confusion and this specific notion of uncertainty are clearly related to each other; however, it can be argued that the confusion concept is less grounded in specific epistemological or pedagogical concerns, and can also be accounted for by the interaction context and lack of clarity in the part of an interlocutor.

In terms of concrete models of confusion, the state-of-the-art is limited in detail but sets a clear framework. Arguel and Lane (2015) proposed two key thresholds (*T*_*a* and *T*_*b*) for the levels of confusion in learning. Productive confusion occurs between these two thresholds, indicating that the learners are engaged in solving their confused state. When the level of confusion is greater than *T*_*b* (persistent confusion), the cognitive states of the learners can move to a state of frustration or even boredom. However, if the level of confusion is less than *T*_*a*, then the learners continue to participate in their learning. Similarly, Lodge et al. (2018) proposed a zone of optimal confusion and suboptimal confusion. Optimal confusion is a productive confusion, indicating that learners are still engaged in overcoming the confused state. However, suboptimal confusion is associated with persistent confusion in which learners could not resolve their disequilibrium, which in turn can lead to possible frustration or boredom. Furthermore, D’Mello and Graesser (2014) proposed a transition-oriented model where confusion can be seen as a part of the emotional transition within the bilateral orientation of engagement/flow and frustration/boredom.

The concept of confusion also has a relationship to the related term “disequilibrium state” and associated concept “cognitive disequilibrium”. Specifically disequilibrium state is a state that arises when individuals encounter obstacles to their goals, interruptions in organized action sequences, contradictions, anomalous events, dissonance, incongruities, unexpected feedback, uncertainty, deviations from norms, and novelty (Graesser et al., 2005; D’Mello and Graesser, 2012; Lehman et al., 2012a). Thus, in many ways a disequilibrium state can be thought of as a state that arises due to a range of obstructing phenomena. The concrete relationship between confusion and disequilibrium, with Lehman et al. (2012a) noting that confusion can be construed as an affective component of cognitive disequilibrium.

Another related concept is that of “metacognitive awareness”. In the education field, metacognitive awareness has been defined as the ability of learners to know when and how to apply knowledge and strategies. Meanwhile, this ability is said to allow individuals to reflect on their self-thinking to develop and apply practical problem solving skills for learning difficulties (Joseph, 2009; Negretti, 2012). In general, Flavell (1979) argued that metacognition is an exclusive human capacity involving self-reflection, monitoring and the governance of one’s own knowledge and thoughts. Metacognitive awareness encompasses three aspects that Negretti (2012) summarized: 1) Declarative knowledge, being aware that specific solutions and concepts are significant relating to a specific task; 2) procedural knowledge, pertaining to understanding how to employ concepts and strategies, essentially the “how” of task execution; and 3) conditional knowledge, which relates to recognizing when and the reason to utilize specific knowledge and strategies, focusing on “when” and “why” aspects of their application. In that study, “metacognitive awareness” is defined as a mental process in which a user becomes aware of the user’s mental state, such as confusion, and subsequently takes action or understands the underlying cause.

Based on the above approaches, and our own earlier attempts to define confusion as something which can be operationalized (Li et al., 2021), we adapt two confusion state definitions on top of which studies can be predicated. We define **productive confusion** as the first stage of confusion. Here, an impasse in the flow of interaction has been generated due to a disequilibrium state. In such a case, a person has reached metacognitive awareness of the confusion, and will generally be involved in solving this disequilibrium. Meanwhile, **unproductive confusion** is the second stage of confusion in which the disequilibrium state is persistent and the impasse cannot be solved directly during the interaction. Here, the interlocutor may become disengaged and may cease interacting with others and may experience negative emotion states (for example, frustration and boredom).

The transition from productive to unproductive confusion is by its nature a change of mental process. When an individual enters productive confusion, they typically attempt to overcome confusion states, but these attempts can of course fail. Reasons for such failure might be because solving this task is beyond the users’ metacognitive awareness and, or, they cannot get any further external help to answer the question, assimilate knowledge, or perform the task in question. At this point, the user may then enter an unproductive confusion state. It is worth noting that the process of solving productive confusion in the aforementioned zone of optimal confusion is closely related to the scaffolding process in the related zone of proximal development (ZPD). Here, a scaffolding process supports the learning environment according to the adaptation of the controlled level that is exercised by a tutor in supporting learners’ understanding (Wood, 2001). The scaffolding support lies in the ZPD, and the ZPD concretely represents the distance between the actual level of the learners and their potential level from Vygosky’s sociocultural theory in educational contexts (Wood et al., 1976; Bruner, 1984).

While the frameworks above provide useful frameworks to anchor an understanding of what confusion is, and how it has been thought about to date in the pedagogy and social science communities, the practical consideration in the context of human-robot interaction must be on manifestations of confusion and their mitigation. In practical interactions with robots in social settings, there is a significant likelihood of confusion, and that any such confusion is at best a negative experience for users, and at worse can be a safety critical challenge. Consequently, there have been some useful trends in research aimed at deliberately inducing confusion states in order to investigate the mechanisms and characteristics linked to confusion.

While there have been no concrete studies in the HRI literature aimed at analyzing the challenge of confusion induction, in the literature, we can identify four patterns of confusion induction (also called confusion cause) and their corresponding non-confusion correlates (Silvia, 2010; Lehman et al., 2012b) which are useful in informing our understanding of confusion in practical task-oriented interactions. The first of these, we refer to as information complexity, which has associated stimuli complex information or simple information. Complex information learning is an experience full of emotions that occurs when learners are exposed to complex material, difficult issues, or indecisive decisions, leading to stimulating their confusion between positive and negative emotions (Lehman et al., 2012b; Arguel et al., 2017). The second pattern is information consistency. Here users might be exposed to contradictory or consistent information where contradictory content is typically associated with participant uncertainty and confusion (Lehman et al., 2013). We refer to the third pattern as information sufficiency. Here participants might be exposed to either sufficient or insufficient levels of information and, based on this, may not be able to understand a concept or perform a task (Silvia, 2010). The final pattern that we identify is incorrect feedback; rather than being focused on the direct ideational content of language as is the case in the first three patterns, this pattern is focused on the interaction and the fact that an interlocutor might provide invalid feedback with respect to expectations following the participant themselves contributing to the interaction. In this pattern, Lehman et al. (2012b) designed a feedback matrix of feedback states that distinguishes between correct feedback, which comprises correct positive conditions and incorrect negative conditions, and false feedback, which includes correct negative and incorrect positive conditions. From their experiment, it was witnessed that the presentation of correct-negative feedback, *i.e.*, when learners responded correctly but received inaccurate or negative feedback was an effective manipulation to stimulate confusion.

### 2.2 Emotion recognition

While studies of confusion directly in the HCI and HRI communities have been limited, there have however been many studies of related phenomena which while not directly capturing confusion, capture phenomena which we argue are closely associated with confusion. The first of these is emotion.

Emotion is a fundamental factor in HRI that affects people’s attitudes and influences their decisions, actions, learning, communication, and situation awareness (Poria et al., 2017; Demutti et al., 2022). If a person has a strong ability to observe others’ emotions and manage their own emotions, they are likely to contribute more successfully to the interaction with others (Poria et al., 2017). Similarly, a social robot is arguably expected to possess human-like capabilities to observe and subsequently interpret human emotions. Building on this idea, Spezialetti et al. (2020) identified three broad sets of tasks that are required to equip robots with emotional capabilities: a) designing emotional states for robots in existing cognitive architectures or emotional models; b) formulating rich emotional expressions for robots through facial expression, gesture, voice, *etc.,*; and c) detecting and inferring human emotions. The first two areas are pure robotics-oriented research, while the last area may be considered a more general HCI consideration which must be tailored to the physically situated nature of the HRI relationship.

While the general principle of identifying and modeling a user’s emotional state seems somewhat straightforward, it should be noted that this is by no means always true. Cohn (2007) argues, for example, that human emotions cannot be observable directly because emotion is a cognitive state which may or may not be related either to physiological and neuromuscular change. Therefore, in practice, an emotion might be explained only through an interaction context and asserted through a user survey. Therefore, to recognize emotions and build emotion models, it is necessary to design specific experiments to trigger a participant’s different emotions from a multimodal learning perspective.

As might be expected, facial features are a very frequently studied modality of emotional expression. Important early work includes the facial action coding system (FACS) with facial action units which is a part-based method that is well known in facial behavior research for the analysis of facial expressions (Cohn, 2007; Menne and Lugrin, 2017). More recently, Canal et al. (2022) summarized two distinct groups of classification algorithms for facial emotion recognition on facial images: classic methods and neural network-based approaches. Classic methods use classical artificial intelligence and image processing such as hand-crafted feature design processing, wherein human experts engineer feature selection to extract a set of features for training the facial emotion recognition model. In contrast to this, the neural approaches focus on the application of Convolutional Neural Networks (CNNs) and generic image processing backbones to build often much more robust emotion detection algorithms. For several years, CNNs have been shown to provide highly accurate results in image analysis in emotion recognition (Refat and Azlan, 2019).

Beyond direct facial expression, image processing has also been used to assess emotion based on other visual features such as head pose Murphy-Chutorian and Trivedi (2009), eye tracking (Mavridis, 2015), and eye gaze (Zhang et al., 2020). Justification for such approaches is exemplified by the work of Emery (2000) who explains that eye gaze is a component of facial expression that is used as a cue to demonstrate the attention of the person to another individual, an event, or an object. In their work, they proved that eye gaze as a special cognitive stimuli is “hard-wired” in the human brain. Moreover, in psychophysiology field, Bagherzadeh-Azbari et al. (2023) showed that the emotion expressions with different gaze direction (including averted gaze and direct gaze) are significant social singles in human interactions.

Emotion recognition is not limited to image-based methods. Speech emotion recognition (SER) has wide real-life applications, including, for example, call centers (Wani et al., 2021), online learning (Cen et al., 2016), spoken dialogue system (Yeh et al., 2019), pain recognition (Özseven, 2019), depression diagnosis (Singh and Goel, 2022), *etc.* Emotion states are identified in a typical SER system from the speech signal without linguistic knowledge (Altun and Polat, 2009). Unfortunately, there are still no common approaches to extract the speech features to a consistent set of specific emotion categories (Singh and Goel, 2022). Nevertheless, Wani et al. (2021) outlines the classes of audio features relevant to emotion analysis, *i.e.*, spectral features, prosodic features, the teager energy operator, and voice-quality features. In recent years, deep learning has also become the dominant field, with architectures such as CNNs, RNNs, LSTMs, and transformers, *etc.*, all being used to automatically learn speech features (Singh and Goel, 2022).

Beyond individual modalities, various recurrent and ensemble network architectures have been built to analyze multimodal datasets, including speech (audio) data, text-based data and video data, and to estimate emotional states (Hazarika et al., 2018; Tripathi and Beigi, 2018). It can be seen that the use of multimodal data offers valuable insight into the domain of emotion recognition within dialogues for both HCI and HRI.

### 2.3 Engagement estimation

While the emotional state of a user is in many ways fundamental, it can often be more beneficial from an HRI perspective to model the user’s engagement. Such research is motivated by the underlying need to ensure that users are motivated to engage and continuously communicate with the robot or system over non-trivial periods. Engagement in social and cognitive psychology is often expressed through one of three aspects: social connection, mental state, or motivated and captivated phenomena (Sidner et al., 2004; Jaimes et al., 2011; Doherty and Doherty, 2018). Of these, probably the most relevant for the HRI perspective is the social connection aspect, where engagement is a process in which participants start to establish a connection, try to maintain this connection, and eventually complete their connection (Sidner et al., 2004; Doherty and Doherty, 2018). Meanwhile for the motivated and captivated phenomena, engagement may not apply to a single interaction but may instead measure a long-term relationship. This is particularly true when engaging with a social platform, although interaction with robotic platforms over time is certainly a long-term desire (Jaimes et al., 2011).

Engagement has been studied with a range of experimental modalities, both within the field of HRI and beyond. In one notable study, Ben Youssef et al. (2017) studied spontaneous conversation with a humanoid robot (a Pepper robot) in a public institute setting. Through four designed conversational interaction sessions, multimodal user data was collected to analyze user engagement against the results of post-study surveys. In another experimental setting, Tapus et al. (2012) looked at four single-subject experiments on social engagement between children with autism and the humanoid Nao robot. This study made use of a wizard-of-oz methodology where two rooms were set up, one for the child and the robot, and one for the operator who controlled the robot’s movements. Even recently, Zhanatkyzy et al. (2023b) designed and empirically investigated 24 robot activities with different levels of social mediation in a rehabilitation setting that included children with different autistic traits. This study was notable in that it used the Nao robot platform to investigate the types of social robot activities that could improve social behaviors for those children with autism.

Regarding the specific detection of engagement, Ben Youssef et al. (2017) presented an analysis of self-reported engagement levels with respect to posture tracking and facial expression analysis. Turning to Tran et al. (2020)’s study in the pedagogy space, the authors measured task precision, reaction time, perceived mental workload, and perceived communicative effectiveness as measures to detect user engagement. Moreover, free initiation in children and robot experiments concerned the gross motor actions that the child performed without prompt while looking at the robot or human interaction (Tapus et al., 2012). Gaze shifting then referred to the occasions spent moving gaze between the robot and the human. While these studies are interesting in that they consider various factors associated with engagement estimation, it is notable that the settings for these studies are often heavily oriented toward pedagogical factors. Few studies within the field of HRI have concentrated on measuring user engagement by combining multiple social behaviors exhibited during dialogues between a human and a robot. This points to a gap in the existing research, emphasizing the need for more comprehensive investigations into the complex dynamics of engagement in HRI.

In this section, while the above studies do indicate that there have been some advances with respect to defining confusion states and the sets of circumstances which can give rise to those states, there has, beyond our own previous research, as of yet, been little done in the way of modeling confusion detection, although there is some work for modeling confusion detection recently in learning, *e.g.*, Atapattu et al. (2020), but not in the field of situated HRI; thus, our studies aim to fill this gap.

## 3 Study design

Below we lay out the overall aims and structure of a new user study which we performed to elicit confusion states in task-oriented HRI. Whereas in an earlier study (Li and Ross, 2023), we induced user confusion and non-confusion states with a range of task types, in this extended study we have narrowed the scope of investigation by focusing on one verbal task type. Moreover, whereas the earlier study considered only the existence of confused and non-confused states, the present study recognizes the proposed existence of three distinct mental states, *i.e.*, productive and unproductive confusion states, as well as the non-confusion state.

### 3.1 Study overview

The general pattern of this study follows our earlier work in Li and Ross (2023) as well as other examples of WoZ style user state analysis studies exemplified by Ben Youssef et al. (2017). Specifically, we again use a controlled methodology where users interact with a humanoid robot platform to perform a series of tasks where some task instances are designed to elicit confusion while others are not. In that pilot study we exposed participants to three different task types, *i.e.*, logical problems, math questions, and word problems. While this was sufficient for demonstrating the detectability of confusion states, this extra dimension of analysis reduces the controllability of the study and makes it impossible in practice to analyze confusion states at a more fine-grained level. In light of this and some other experimental considerations, we redesigned our study to focus on one task type only, *i.e.*, word problem, and redesigned several additional aspects of the study to provide more control and experimental clarity. We also upgraded our experimental devices to collect higher quality multimodal data which we believe will be of higher value to the community.

Specifically, for this study we again used the Pepper robot platform. The Pepper robot includes customizable social behaviors including natural and expressive movements using 20 degrees of freedom, speech recognition in multiple languages, which we configured to English for the current study. Beyond the speech modality, the Pepper platform can observe and interact with a person who is close to it through 2D and 3D cameras onboard, touch sensors, and LEDs for multimodal interactions. For our experiments, we made use of the Naoqi framework, which provides a fully open programmable platform for us to manually control the Pepper robot and design its animated speech and multi-interactive behaviors programmatically and interactively.

**FIGURE 1:**
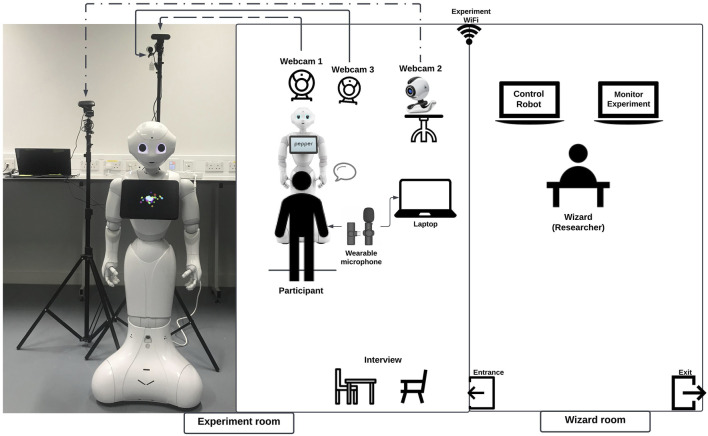
Wizard-of-oz experiment setup (left: the actual experiment room; right: the schematic experiment room and wizard room).

The experimental design is based on a semi-spontaneous physical face-to-face conversation in English between the Pepper robot and each participant. Following registration, a comprehensive introduction of the experiment was shared first with each participant, before consent forms were signed, and the experiment itself began. Each participant was then guided into an experiment room where they were left in front of the Pepper robot. In addition to the robot, the room was equipped with a number of other cameras and microphones to provide extra pickups on the interaction. Since this was a fully WoZ driven study, an experimental controller was set up in a second adjacent room where views of the feeds from the experimental room were available, as well as the controls for the robot itself. Figure 1 presents an overview of this experimental layout, two rooms are isolated, including the experiment room where the participants and the Pepper robot interacted and the wizard room where the researcher controlled the pepper robot and monitored the experiment room. The additional recording equipment included three high-definition webcams. Webcam 1 was placed behind the Pepper robot and oriented toward the participant’s face to clearly monitor their facial expression. Webcam 2 was placed right beside the robot to observe the participant’s body gestures. Finally, Webcam 3 was placed close to Webcam 1, but oriented so that the wizard could monitor the whole process of the experiment from the wizard’s room. Participants were required to wear a lapel microphone and stand along a line that was approximately 80 cm in front of the robot to ensure that they were safe but still in the interactive zone. The equipment and the Pepper robot in both rooms are interconnected through the private experimental network. The picture to the left of Figure 1 shows the actual scene of the experimental setting.

Each interactive session with the Pepper robot had two distinct conversations. The first conversation was a free talk of around 5 min, where participants adapted to interaction with the Pepper robot and the general principles of HRI interaction. For this, the Pepper robot was in full autonomous mode; and during the free talk, a researcher assisted and encouraged participants to engage with the Pepper robot in the experiment room. Moreover, we suggested 11 simple short conversational tasks for participants to engage in interactively with the robot. These included asking the robot, *“Who are you?“, “Can we shake hands?“, “Show your left hand.“*, *etc.* The second conversation was the experimental task-oriented dialogue session, which took approximately 20 min. During this period, the Pepper was changed to be fully controlled by the researcher, and the researcher themselves stayed in the wizard room leaving the participant alone with the Pepper robot in the experiment room. This experimental conversation included eight crafted dialogues based on a verbal problem task (detailed in Section 3.2). After each of the eight dialogues in this conversational interaction, the participants were asked to rate their level of confusion during a 1-min break. Once completed this second conversational session, the researcher returned to the experiment room again and the participants were asked to complete a post-study survey which included a number of statements referenced against a 5-point Likert score. This survey included questions on general experimental HRI experiences, as well as the PARH (Perceived Awareness of the Research Hypothesis) scale (Rubin, 2016) that allow researchers to assess the potential influence of demand characteristics (*i.e.*, “users’ confusion levels”) in this study. Finally, a 3-min oral interview was conducted to collect more feedback on the interaction.

Participants in this study were recruited across a metropolitan university. In total there were 81 individuals (female: 36, male: 44, non-binary: 1) who were each over 18 years of age, and with 21 nationalities represented. Of these, 38 participants were in the 18 − 24 age group, 31 participants were in the 25 − 44 age group, while the 45 − 59 age group had 8 people, and 4 people over 60 years of age. In terms of social profile, 61 people were students at colleges, 14 people worked in academia, and 6 people worked in industry. Participants were not required to have English as a first language, although 45 people were native English speakers. It should be mentioned that only 1 of 81 participants had experience interacting with a humanoid robot. The 81 participants agreed to make their data available for analysis and publication for research purposes.

### 3.2 Task-oriented dialogue design

As indicated, the second part of the main interaction consisted of eight individual dialogues which were broken up by 1 minute breaks. Each of these dialogues was used to express one single experimental condition. In total we had three experimental conditions, or rather stimuli classes, and these correspond to the levels of non-confusion, productive confusion, and unproductive confusion previously introduced. Each individual task was indicated on the basis of a word problem. For notation purposes, the three conditions are referenced as follows, Condition A1 = productive confusion; Condition A2 = unproductive confusion; while Condition B is referred to stimuli which are in principle non-confusing.

In order to limit the potential of confusion persistence and intermingling between experimental conditions, we adopted a cross participant approach to the experimental design, where participants were grouped into either productive or unproductive confusion experimental groups. For the productive confusion experimental group (A1), each participant was first exposed to 4 instances of non-confusion stimuli, before being exposed to a sequence of 4 productive confusion stimuli. Similarly, for the unproductive confusion experimental group (A2), participants were first exposed to 4 instances of non-confusion stimuli, before being exposed to a sequence of 4 unproductive confusion stimuli. Thus, the sequence of eight dialogues for each participant is either “B-B-B-B-A1-A1-A1-A1” or “B-B-B-B-A2-A2-A2-A2” depending on the experimental group to which they were assigned. We refer to these two experimental groups as either **Condition BA1** or **Condition BA2** as appropriate; (see Table 1).

**TABLE 1:** Example of sequences of confusion induction in participants.

Participant 1		
Dialogues	Non-, Confusion Stimulus Type	Conditions
1st	Simple information	Condition B
2nd	Consistent information	Condition B
3rd	Sufficient information	Condition B
4th	Correct feedback	Condition B
5th	Confusion Cause (CC)1*	Condition A1
6th	CC2*	Condition A1
7th	CC3*	Condition A1
8th	CC4*	Condition A1

Participant 2		
Dialogues	Non-, Confusion Stimulus Type	Conditions
1st	Simple information	Condition B
2nd	Consistent information	Condition B
3rd	Sufficient information	Condition B
4th	Correct feedback	Condition B
5th	CC1*	Condition A2
6th	CC2*	Condition A2
7th	CC3*	Condition A2
8th	CC4*	Condition A2

*CC1: Complex information.

*CC2: Insufficient information.

*CC3: Contradictory Information.

* CC4: False feedback.

In previous work, we have used alternative designs which were based on an interleaving of confusing and non-confusing stimuli. While it is likely that our current design introduces its own challenges, we feel that this design strikes the right balance on control with a smaller chance of confusion states, or rather behavioral indicators of confusion states, leaking into states where no confusion should be present. Finally, it should be noted that the non-confusing dialogues, *i.e.*, those labeled Condition B, were identical regardless of being used for either Condition B_A1 in the productive confusion experimental group or Condition B_A2 in another experimental group.

Beyond the three main conditions of non-confusion (B), productive confusion (A1) and unproductive confusion (A2), one further conditional subclassification was introduced into the experimental design, and this relates purely to Condition A1. Namely, the dialogues of Condition A1 were designed to include assistance provided by the Pepper robot to help participants overcome their confusion state. Being more specific, the dialogues were designed such that the robot asks the participant whether they need help to answer the question after the participant has tried to answer the question twice incorrectly or the participant may request a help to the Pepper robot. The interaction up to this assistance request is labeled Condition A1_beforehelp, whereas all interactions thereafter are labeled Condition A1_withhelp. This assistance, in Condition A1_withhelp, involved the robot deliberately moderating its speech pace, presenting the problem step by step, and soliciting feedback from the participant to gauge comprehension. Such differentiation helps us to explore whether there are detectable states for participants as they transition from a confused state to one where that confusion is being overcome. Some participants were able to answer the confusion question without this help from the robot. In such cases, we labeled the entire dialogue sequence as Condition A1_beforehelp. However, in the unproductive confusion condition (A2), the Pepper robot only repeated the question with the same speed of speech until the participant showed their wish to give up.

To attempt to ensure an invocation of confusion that might correspond to the many different potential causes of confusion in practical tasks, we mapped each of the four individual confusion dialogues (either A1 or A2) to one of the four patterns of confusion which we outlined in the previous section. Thus, each dialogue was designed using either one stimulus type designed to induce confusion or a stimulus type designed not to induce confusion (see Table 1). To illustrate the specific nature of the tasks and text used by the robot, eight dialogue scripts based on the four confusion patterns have been included in the resources associated with this paper^2^. It should be noted that while four distinct confusion causes are used across the dialogues, the experimental design, which is different from our previous study, is limited to verbal problems only.

### 3.3 Data collection

During interactions, data were recorded across the Pepper robot’s built-in sensors as well as the higher-fidelity devices that were situated around the experimental room. Media data and post-study survey data were thus collected from 81 participants—41 participants were in the productive confusion experimental group, while 40 participants were in the unproductive confusion experimental group. The multimodal data streams were initially labeled as a whole based on the collection condition, *i.e.*, “Condition BA1” (including Condition B_A1, Condition A1_beforehelp, and Condition A1_withhelp), or “Condition BA2” (including Condition B_A2 and Condition A2), before *post hoc* fine-grained labeling was applied to specific extracts of the data as appropriate. It should be noted that Condition B_A1 and Confusion B_A2 represent the same experimental state in practice, *i.e.*, the extracts of interactions at the start which consist of non-confusion states only. We thus would expect that the two populations should demonstrate similar characteristics and could in principle be grouped together as a single B set in principle. While this is true we have kept these two subgroups of B distinct in analysis as in many cases our interest is in comparing intra-subject behavior between the participants in B_A1 and A1 and B_A2 and A2.

Basic editing as well as some limited preprocessing were applied to the raw data to prepare a dataset for analysis. In each facial video, we cropped the greeting and conclusions, and then extracted facial frame data with conditions labeled. To ensure that each frame instance is a valid facial image, we applied the Multitask Cascaded Convolutional Neural Network (MTCNN)-based face detection algorithm (Savchenko, 2021) to detect the face and then automatically center-crop to a region of 224 × 224 pixels. The resultant set included 28,938 facial frames for Condition A1 (13,742 facial frames for Condition A1_beforehelp, and 15,196 facial frames for Condition A1_withhelp), 26,631 facial frames for Condition A2, and 11,218 facial frames for Condition B (5,695 facial frames with Condition B_A1, and 5,523 facial frames from Condition B_A2).

As for the audio stream, we again removed the greeting and conclusions and cropped several pieces of audio corresponding to five experimental conditions segments of interest in the interaction. After verifying these audio stream data, we had collected 316 audio samples for Condition A1 (162 audio samples for Condition A1_beforehelp, and 154 audio samples for Condition A1_withhelp), as well as 152 and 308 audio samples respectively for Condition A2 and Condition B (156 audio samples labeled with Condition B_A1, and 152 audio samples labeled with labeled Condition B_A2), respectively.

## 4 Data analysis

To investigate participant behavior in all three conditions, we grouped our two objective data types, *i.e.*, visual and audio data, and then applied a range of feature extraction algorithms before analyzing the interactions between those extracted features and both the experimental conditions and the self-assessed confusion levels from the post-study survey. To this regard, we can learn the correlations between each result of the feature analysis and the experiment conditions. In the following, we motivate specific hypotheses for analysis, break down the analysis process, and then provide an overview of the results of this analysis.

### 4.1 Visual data analysis

Our visual investigation was focused on the face, where we borrowed methodologies for learning nonverbal interacting user behaviors in the studies of emotion estimation and user engagement with respect to eye gaze and head pose features.

#### 4.1.1 Facial emotion detection

Given an assumed link between emotional states and confusion (D’Mello and Graesser, 2014; Liu et al., 2022), we applied a facial emotion detection algorithm to estimate for each frame an emotional quotient for each individual frame in terms of common emotion categories, *i.e.*, anger, disgust, fear, sad, happy, surprise, and neutral. While the link between emotion and learning state is well argued in the literature, there are a number of presumptive hypotheses based on previous works that we hoped to be true. Most significantly, we expect that participants in either a productive confusion state or unproductive confusion state demonstrate enter into a more negative emotional state and that this negative state should be apparent from image data. As a corollary we expect that more positive data would be associated with non-confusion states. We similarly expect that the product confusion state should be associated with less negative emotions than the unproductive confusion state—particularly with respect to the with help portion of the analysis.

For the facial emotion detection, we used a model based on the MobileNet architecture and trained in the AffectNet dataset (Howard et al., 2017; Mollahosseini et al., 2017; Savchenko, 2021). The predicted emotion scores are from seven emotion indexes, *i.e.*, 0: “Anger”, 1: “Disgust”, 2: “Fear”, 3: “Happy”, 4: “Neutral”, 5: “Sad”, and 6: “Surprise”. Thus, each image is assigned an emotion type based on these predicted scores. Table 2 shows the results analyzed for both the productive confusion experimental group and the unproductive confusion experimental group. As the dialogue interaction with any confusion condition is longer than these with non-confusion condition, it appears that the number of each detected emotion for Condition B_A1 or Condition B_A2 is less than these for Condition A1 or Condition A2, respectively. Moreover, concerning Condition A1_beforehelp and Condition A1_withhelp, the number of emotions “happy”, “surprise”, and “neutral” in Condition A1_withhelp are greater than these emotions in Condition A1_beforehelp; meanwhile, the number of each of the emotions “anger”, “fear” and “sad” in Condition A1_beforehelp is greater than those in Condition A1_withhelp, excluding the predicted emotion “disgust” that is ignored due to the limitations of the facial emotion algorithm for these results (Li and Ross, 2023).

**TABLE 2:** The result of facial emotion estimation in Condition A1, A2, and B.

Condition (labeled)	Anger	Disgust	Fear	Sad	Happy	Surprise	Neutral	Overall
A1_beforehelp	3,110	1,503	95	2,693	1,961	81	4,299	13,742
A1_withhelp	3,020	1,877	90	2,680	2,192	136	5,201	15,196
B_A1	1,086	826	37	1,025	646	35	2,040	5,695
A2	4,820	2,411	304	4,910	7,173	331	6,682	26,631
B_A2	815	495	51	949	1,431	50	1,732	5,523

While facial emotion recognition algorithms are beneficial in many tasks, it is known that they may still produce imperfect results at fine granularities. Hence, following the 2-D model that integrates valence and arousal (Russell, 1980), we grouped the seven predicted emotions into four broader categories based on the individual’s mental state, *i.e.*, negative emotion that includes “sad”, “fear”, “anger”, and “disgust”, positive emotion which is a singleton class that includes only the “happy” emotion, the surprise emotion (Vogl et al., 2019), and finally the neutral emotion. Moreover, following (Maithri et al., 2022) who shows that these boarder emotion categories can be modeled in three dimensions, namely, valence-arousal-dominance. This modeling can help to assess the level of stimulus control.

**FIGURE 2:**
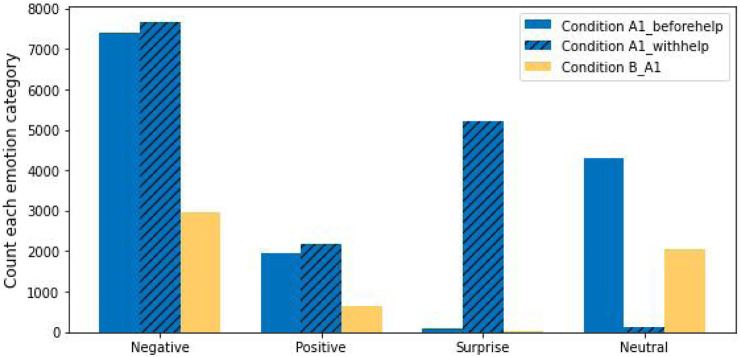
Four facial emotion categories for Condition A1 and Condition B.

Considering first the case for the data with Condition A1, these results are presented in Figure 2. We see that the number of negative instances, positive instances and surprise instances within Condition A1_withhelp are greater than these emotion instances within the other two conditions; while for Condition A1_beforehelp the number of neutral emotions is greater than those with the other two conditions. Finally, it should be noted that the number of surprise emotion instances is almost 0 in both Condition B_A1 and Condition A1_beforehelp. We suggest that these results indicate that participants were more engaged in answering the question with the Pepper’s help.

**FIGURE 3:**
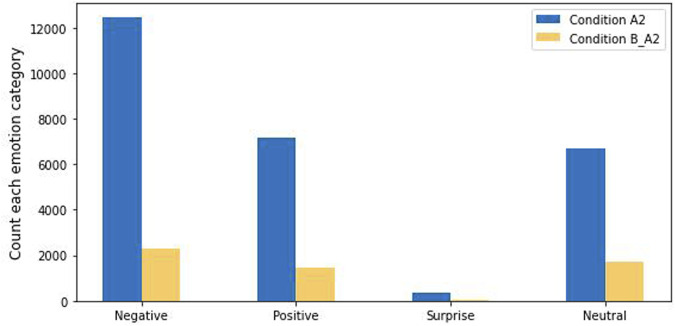
Four facial emotion categories for Condition A2 and Condition B.

Turning to the results for Condition A2, for the four emotion groupings classified in Condition A2 and Condition B_A2, the number of negative instances, positive instances, and surprise instances in Condition A2 is greater than that of Condition B_A2—see Figure 3. We suggest that these results show that, without the robot’s help, the participants were more emotionally expressive and taking longer interactions in the word problem tasks in Condition A2 than in Condition B.

To provide a more detailed analysis, as the number of each emotions in Condition A1_beforehelp, Condition A1_withhelp and Condition A2 are greater than in Condition B_A1 and Condition B_A2, but yet those predicted data were still a small size, we randomly selected sub-results of facial emotion predicted outputs in Condition A1_beforehelp, Condition A1_withhelp and Condition A2 which are the same number of those outputs in Condition B_A1 and Condition B_A2 respectively, for statistical analysis. Across productive confusion and non-confusion, a Chi-Square test for independence (with Yates’ Continuity Correction) indicated a significant association between seven emotion indices and our three labeled conditions (Condition A1_beforehelp, Condition A1_withhelp and Condition B_A1), ${\tilde{\chi }}^{2}\left(1,\;n=34,633\right)=\left.169.83,p-value<0.05,phi=0.07\right)$ . Moreover, a Chi-Square test for independence (with Yates’ Continuity Correction) indicated significant association between the seven emotion indices and two labeled conditions (Condition A1_beforehelp and Condition A1_withhelp), ${\tilde{\chi }}^{2}\left(1,\;n=28,938\right)=82.45,p-value<0.01,phi=0.05$ ). Turning to the emotion estimation results across unproductive confusion and non-confusion states, a Chi-Square test for independence (with Yates’ Continuity Correction) indicated significant association between our seven emotion indices and the two conditions (Condition A2, Condition B_A2), ${\tilde{\chi }}^{2}\left(1,\;n=32,154\right)=\left.110.02,df=6,p-value<0.01,phi=0.06\right)$ .

**FIGURE 4:**
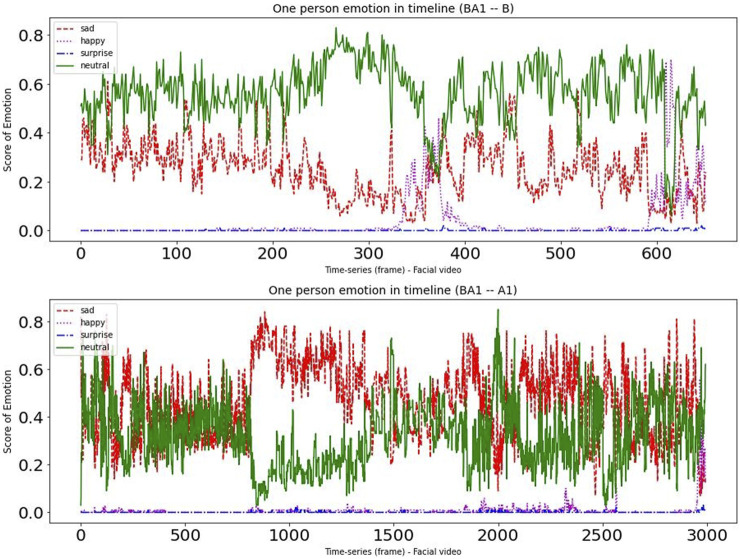
The emotional changes for one participant during dialogues with conditions (BA1).

**FIGURE 5:**
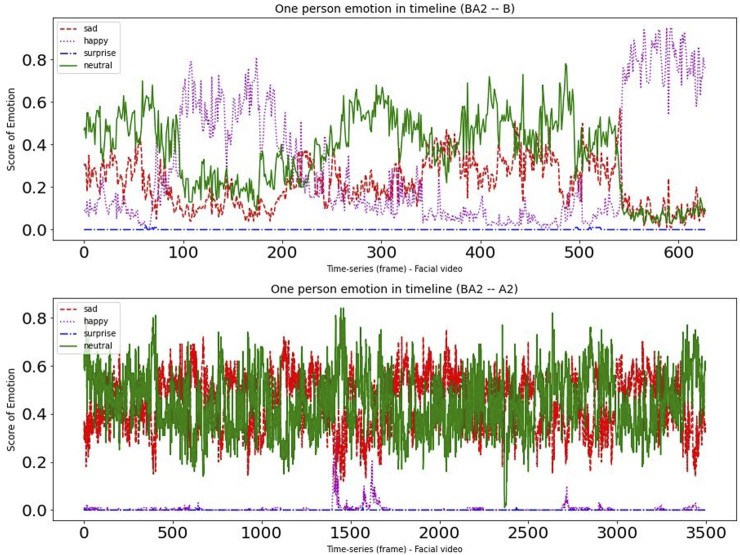
The emotional changes for one participant during dialogues with conditions (BA2).

To provide deeper insight into the data, we randomly chose one participant from each of the two experimental groups. Figures 4, 5 present a time-series visualization of the emotional group (four states, *i.e.*, “happy”, “sad”, “surprise” and “neutral”) for two participants for each of the two experimental groupings in the facial videos. For this particular analysis, we used the open source facial expression recognition application (FER)^3^. The FER application is built on the MTCNN facial recognition network (Zhang et al., 2016), with the emotion classifier (Arriaga et al., 2017) trained on the FER-2013 emotion dataset (Goodfellow et al., 2013). In the productive confusion experimental instance we can see that “neutral” and “sad” occurrences dominate, while the “happy” emotions can sometimes climb to the top—particularly during the non-confusion periods corresponding to Condition B. Meanwhile, in the unproductive confusion group participant, we see “neutral” and “happy” dominated during Condition B, while for Condition A2 the emotions “sad” and “neutral” were very strong.

#### 4.1.2 Eye gaze estimation

Moving from facial emotion to eye gaze estimation, the importance of eye gaze and its impact on emotion and engagement levels has been previously investigated, and based on the literature we hypothesize that the range of eye gaze for users with either productive confusion or unproductive confusion would be less than for those participants in a non-confusion state.

To investigate this, we applied a state-of-the-art eye gaze estimation algorithm, trained on the large-scale gaze estimation dataset called ETH-XGaze (Zhang et al., 2020), to our pre-processed facial frame data, in order to predict pitch and yaw angles. Since different angles may have positive or negative values, a composite sum of angles for a given user would likely cancel out to zero; therefore, as a composite metric, we instead summed the two absolute angles to produce an amalgamated metric for each user.

**FIGURE 6:**
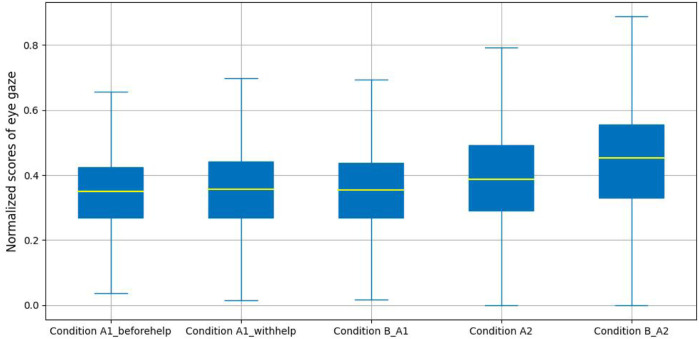
Eye gaze estimation analysis. The line in yellow represents the median.

Figure 6 presents the amalgamated metric (absolute yaw and pitch) across our experimental conditions. From these results we can see that the median of the composite feature for participants in Condition B_A2 is greater than that of the other four labeled conditions. However, there is a notable, though slight difference in the median of the composite between Condition A1_beforehelp, Condition A1_withehelp and Condition B_A1.

To provide a more detailed analysis, we applied a one-way ANOVA to explore the impact of normalized the sum of absolute pitch and yaw angles for eye gaze with experimental conditions including productive confusion and non-confusion. A significant difference was found in the angle scores (*i.e.*, normalized sum of absolute pitch and yaw angles) for eye gaze across Condition A1_beforehelp, Condition A1_withhelp and Condition B_A1, (*F*(2, 29784) = 14.61, *p* − *value* < 0.01, *d* = 9.80*e* − 04). Tukey’s HSD (Honestly Significant Difference) test for multiple comparisons found that the mean scores in Condition B_A1 (*M*=0.36, SD = 0.13) were not significantly different from those in Condition A1_beforehelp (*M* = 0.35, SD = 0.13), *p* − *value* = 0.36; however, the mean scores for those in Condition A1_withhelp (*M* = 0.36, SD = 0.14) were significantly different from those for Condition A1_beforehelp *p* − *value* < 0.01, as was the case for Condition B_A1, *p* − *value* < 0.05. Regarding unproductive confusion, we again calculated the normalized sum of absolute pitch and yaw angles as a composite feature, and an independent sample t-test showed that there was a significant difference in this composite value across the two labeled conditions (*M* = 0.40, SD = 0.15 for Condition A2, *M* = 0.45, SD = 0.16 for Condition B_A2), *t*(26736) = −21.205, *p* − *value* < 0.01, *d* = −0.26.

#### 4.1.3 Head pose estimation

Similar with eye gaze, head pose has been found to have correlations to engagement and emotion level, and based on this we hypothesized that the ranges of head pose for participants in either productive confusion or unproductive confusion states should be less than those of users who are in non-confusion states.

To test this, on our aligned facial images, we applied a trained head pose estimation model based on a CNNs backbone, dropout and adaptive gradient methods which was trained on three public datasets: the Prima head pose dataset, the Annotated Facial Landmarks in the Wild (AFLW), and the Annotated face in the Wild (AFW) dataset (Gourier et al., 2004; Köstinger et al., 2011; Zhu and Ramanan, 2012). From this trained model we extracted the three angles of pitch, yaw, and roll for each image frame. These values were then grouped across our five labeled conditions. Similarly to the analysis of eye gaze estimation, we calculated the sum of the three absolute values of these angles to produce a new composite feature.

**FIGURE 7:**
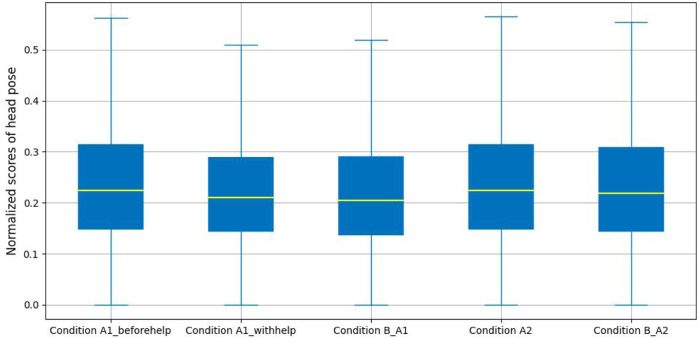
Head pose analysis.

Figure 7 demonstrates a slight difference between the head pose estimation results for the five labeled conditions. From these results we can see that the median composite value for Condition B_A1 and Condition B_A2 is lower than that in Condition A1 (Condition A1_beforehelp and Condition A1_withhelp) and Condition A2, respectively. Meanwhile, the median value in Condition A1_beforehelp is higher than those for Condition A1_withhelp.

Again, similar to the eye gaze estimation, we performed a statistical analysis of these results by applying a one-way ANOVA which showed that a significant difference was found in the composite angle scores (*i.e.*, normalized sum of absolute pitch, yaw and roll angles) measurement across Conditions A1_beforehelp, Condition A1_withhelp and Condition B_A1 (*F*(2, 34622) = 32.08, *p* − *value* < 0.01, *d* = 1.85*e* − 03). Tukey’s HSD test for different comparisons of any two conditions illustrated that the mean scores for Condition A1_beforehelp (*M* = 0.25, SD = 0.14) was significantly different from those for Condition A1_withhelp (*M* = 0.24, SD = 0.13), *p* − *value* < 0.01 and also significantly different for Condition B_A1 (*M* = 0.23, SD = 0.14), *p* − *value* < 0.01. However, there were no significant differences for the mean head pose scores between Condition A1_withhelp and Condition B_A1, *p* − *value* = 0.98. For the unproductive confusion experimental group, an independent sample t-test was performed showing that there was a significant difference for the composite angle scores across (*M* = 0.25, SD = 0.14 for Condition A2, *M* = 0.24, SD = 0.14 and Condition B_A2), *t*(32152) = 2.78, *p* − *value* < 0.01, *d* = 0.03.

### 4.2 Audio data analysis

With respect to audio data, our focus is on the features of speech that can be taken as indicators without requiring a full semantic or context-specific analysis of the speech. Thus, in this section, we present the analysis of low-level features including silence time, speech emotion recognition, acoustic feature vectors, *e.g.*, Mel-Frequency Cepstral Coefficients (MFCCs) feature (Mohan et al., 2023), as well as pitch of speech.

#### 4.2.1 Emotional silence duration analysis

Silence or delayed response in a dialogue is a useful feature of nonverbal interaction. Oto et al. (2017) suggested that the silence in HRI can be divided into four types, *i.e.*, semantic silence, syntactical and grammatical silence, robotic silence, and interactive silence, and that interactive silence may at times reflect the user’s strong emotion such as anger, surprise, and fear, *etc.* Broadly speaking, we can therefore hypothesize that the emotional silence duration of the participants in a non-confusion condition should be shorter than that for participants in either the productive confusion or unproductive confusion condition.

Based on this hypothesis, in our analysis of the audio data, we accounted for interaction silence by labeling and subsequently measuring the silence duration time of the participants only between the point after the robot asked a question or gave feedback to the participant, but before the participant started to respond orally. Then the labeled silence duration time was normalized under the different experimental conditions.

**FIGURE 8:**
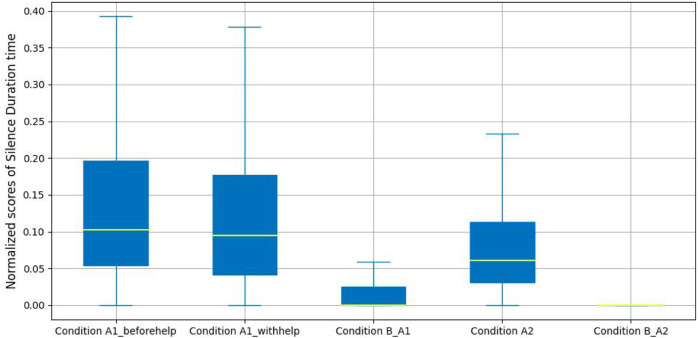
Silence duration time analysis.

Figure 8 presents a comparison of the normalized silence duration time for five labeled conditions. From these results we can see a sharp difference in silence duration time between confusion conditions and non-confusion conditions. The median score of the silence duration time is 0 in Condition A2_B and Condition A1_B; and there is a slight difference in the median scores between Condition A1_beforehelp and Condition A1_withhelp. Meanwhile, the median score of the silence duration time in Condition A2 is lower than that for both labeled conditions in productive confusion. We suggest that this demonstrates that the participants expected to have to wait for the robot’s help in order to overcome their confusion in dialogues with productive confusion.

For statistical analysis, a one-way ANOVA was performed to investigate the impact of the interactive silence duration across productive confusion and non-confusion periods. There was a significant difference in the normalized silence duration time across Condition A1_beforehelp, Condition A1_withhelp, and Condition B_A1 (*F*(2, 660) = 40.56, *p* − *value* < 0.01, *d* = 0.11). Tukey’s HSD test found that the mean scores for Condition B_A1 (*M* = 0.04, SD = 0.11) were significantly different from those for Condition A1_beforehelp (*M* = 0.15, SD = 0.16), *p* − *value* < 0.01 and also differ significantly for Condition A1_withhelp (*M* = 0.13, SD = 0.15), *p* − *value* < 0.01; however, the mean scores for Condition A1_beforehelp were not significantly different from those for Condition A1_withhelp, *p* − *value* = 0.35. Turning to the unproductive confusion experimental group, an independent sample t-test was performed, indicating that there was a significant difference between the normalized silence duration time across the two conditions (*M* = 0.09, SD = 0.10 A2, *M* = 0.02, SD = 0.08 and B_A2), *t*(990) = 12.57, *p* − *value* < 0.01, *d* = 0.14.

#### 4.2.2 Emotional pitch analysis

Pitch is one of the prosodic elements in speech signals, and is also a nonverbal parameter in human social communications, whereby various expressions of emotions can be reflected by changes in different ranges of pitches, *e.g.*, people in a happy state can be detected by higher pitch and larger range of pitch, whereas, when people feel sad, their speech signals are typically slower with lower average pitch and narrower range of pitch, *etc.* (Gasteiger et al., 2022). Based on this potential relationship to emotional state, and hence an indicator of more pronounced confusion states, we can express a broad hypothesis that the emotional pitch of the participants in a non-confusion state should be higher than that for participants in either the productive confusion or unproductive confusion states.

**FIGURE 9:**
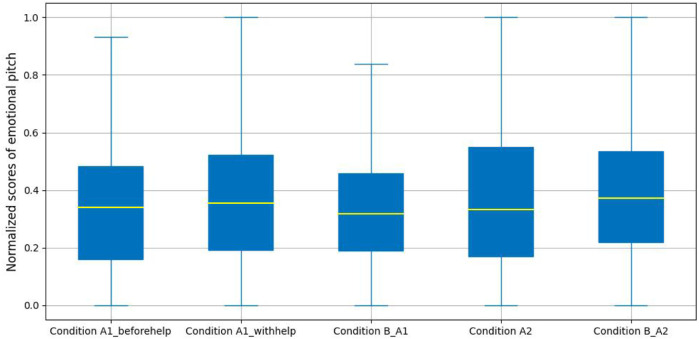
Emotional pitch analysis.

In our analysis, we extracted pitch values using the YAAPT (Yet Another Algorithm for Pitch Tracking) pitch tracking algorithm (Zahorian and Hu, 2008). As the pitch results of each audio sample are a sequence of temporal pitch values, we calculated the mean value of each set of values as a new feature (called pitch value) for analysing. Figure 9 presents a detailed comparison of the mean pitch value for the five labeled conditions. It shows that there are minor differences in each experimental group. The median of pitch values in Condition A1_B is slightly lower than that in Condition A1 (*i.e.*, Condition A1_beforehelp and Condition A1_withhelp). Whereas, in the unproductive experimental group, the median of pitch values in Condition A2_B is greater than that in Condition A2.

To further analyze, for the productive confusion experimental group, we conducted a one-way ANOVA on the YAAPT estimations to explore the influences of normalized user pitch values under the productive confusion conditions. We found that there was no significant difference in normalized pitch values with Condition A1_beforehelp, Condition A1_withhelp, and Condition B_A1 (*F*(2, 470) = 1.22, *p* − *value* = 0.30). For the unproductive confusion experimental group, an independent sample t-test was performed. Again, no significant differences were found for the normalized pitch values between the two conditions (*M* = 0.39, SD = 0.25 for Condition A2, *M* = 0.39, SD = 0.37 for Condition B_A2), *t*(302) = −0.27, *p* − *value* = 0.79.

#### 4.2.3 Speech emotion analysis

Speech signals can also be used to directly infer an associated emotional signal, which again may have a direct influence on confusion states as per the argument set forward for visual indicators. On that basis we again hypothesize that participants in a non-confusion state will demonstrate more positive emotions as measured from speech signals directly than is the case for participants in a confusion condition.

To investigate this possibility, we cropped all participant audio speech signals for each labeled condition, then extracted the speech spectral features from those data using MFCCs features as input, in order to directly predict four different salient emotion indexes, *i.e.*, “anger”, “happy”, “neutral”, and “sad”. For this, we used the trained Temporal-aware bI-direction Multiscale Network (TIM-Net) model (Ye et al., 2023), which is a state-of-the-art temporal emotional modeling solution, trained on six benchmark Speech Emotion Recognition (SER) datasets, *i.e.*, Chinese corpus CASIA, German corpus EMODB, Italian corpus EMOVO, English corpora IEMOCAP, RAVDESS and SAVEE (Busso et al., 2008; Tao et al., 2008; Jackson and Haq, 2010; Costantini et al., 2014; Livingstone and Russo, 2018; Benlamine and Frasson, 2021).

**FIGURE 10:**
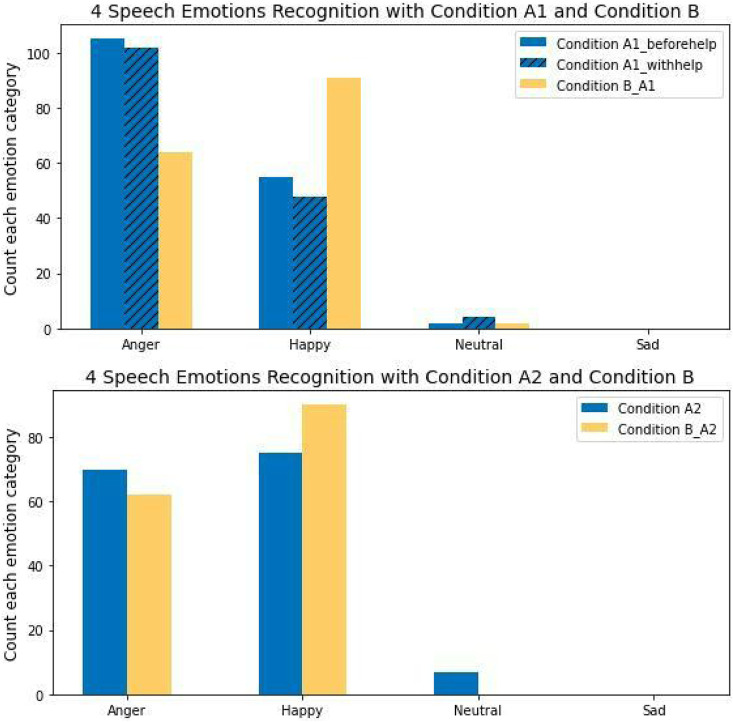
Four emotions speech recognition.

Figure 10 presents the predicted emotion categories with respect to the two experimental groups. Notable results include the fact that there were no instances of the “sad” emotion in the participants’ speech, and that the number of “happy” instances in Condition B_A1 was greater than those of the other two labeled conditions. Additionally, the number of “anger” instances recorded for Condition A1_beforehelp was greater than those in both Condition A1_withhelp and Condition B_A1. Meanwhile, the number of “neutral” instances in Condition A1_withhelp was slightly higher than that of Condition A1_beforehelp and Condition B_A1. For the unproductive confusion experimental group, the number of “happy” instances in Condition B_A1 was greater than in Condition A2, while the number of “anger” emotions in Condition B_A1 was less than that for Condition A2. Finally, it should be noted that the number of “neutral” instances in Condition B_A2 was 0, while there were no “sad” instances recorded for user speech in either labeled conditions.

In the statistics analysis of emotion speech, it suggests that participants were on average happier in dialogues with non-confusion condition than those with either confusion state. A one-way ANOVA was used to investigate the impact of emotion speech with productive confusion and non-confusion. We found that there was a significant difference in the normalized emotion scores across Condition A1_beforehelp, Condition A1_withhelp, and Condition B_A1(*F*(470) = 6.30, *p* − *value* < 0.01, *d* = 0.03). Tukey’s HSD test found that the mean emotions scores for Condition B_A1 (*M* = 0.27, SD = 0.18) were significantly different from those for Condition A1_withhelp (*M* = 0.19, SD = 0.19), *p* − *value* < 0.01, but did not significantly differ for Condition A1_beforehelp (*M* = 0.25, SD = 0.23), *p* − *value* = 0.54. Moreover, there was a significant difference in mean emotion scores between Condition A1_beforehelp and Condition A1_withhelp, *p* − *value* < 0.05. For the unproductive confusion experimental group, an independent sample t-test was performed which showed that there was a significant difference between the normalized emotion scores in users’ speech between Condition A2 (*M* = 0.31, SD = 0.27) and Condition B_A2 (*M* = 0.41, SD = 0.25), *t*(302) = −3.48, *p* − *value* < 0.01, *d* = −0.4.

### 4.3 Self-reporting analysis

In addition to analyzing the results of raw audio and video data, we also present an analysis of the participant’s self-reported confusion scores and related subjective estimates with confusion states on the Likert scale (1–5). In particular, we assess whether there is a significant difference in user-reported confusion levels with respect to the experimental conditions; and second, whether the participants tried to push through the confusion or whether the participants wanted to stop in order to otherwise resolve their unproductive confusion; third, whether the mean PARH scores are significantly below the scale midpoint 4).^4^ Broadly we expect that self-reported confusion scores should be higher for participants in the confusion state than for those in a non-confusion state.

**FIGURE 11:**
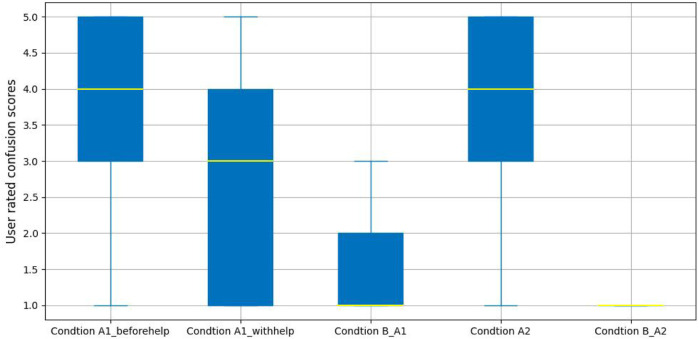
User reported confusion levels analysis.

Figure 11 presents the results for the self-reported confusion estimates. From these results, we can see that the median confusion scores is 1 in both non-confusion conditions. The highest median confusion scores were 4 in Condition A1_beforehelp and Condition A2, and the median of confusion scores in Condition A1_withhelp was 3, which is in the middle of confusion levels. These results suggest that there is a very highly pronounced change in self-reported confusion levels that corresponds to the expectations of the experimental conditions.

For the productive confusion experimental group, a Kruskal-Wallis rank sum was performed to explore the impact of users confusion scores with those labeled conditions, and a significant difference was found in confusion scores for Condition A1_beforehelp (*Mdn* = 4.00, *M* = 3.79, SD = 1.27), Condition A1_withhelp (*Mdn* = 3, *M* = 2.68, SD = 1.44), and Condition B_A1 (*Mdn* = 1.00, *M* = 1.50, SD = 0.85), ${\tilde{\chi }}^{2}\left(2\right)=174.47,p-vlaue<0.01,H=0.36$ . For the unproductive confusion experimental group, a Mann-Whitney U test was performed, showing that the confusion scores with Condition A2 (*Mdn* = 4.00, *IQR* = 2.00) were significantly higher than those with Condition B_A2 (*Mdn* = 1.00, *IQR* = 0.00), *U* = 2, 327, *Z* = −13.27, *p* − *value* < 0.05, *r* = 0.74.

To examine whether the participants are aware of productive confusion or unproductive confusion, after each task-oriented dialogue, a few more questions were asked of each participant (more details are provided in the surveys). For the productive confusion conditions, the proportions of responses to the question of whether users solved the task with the robot’s help were: 69% “Yes, resolved”, 26% “No, I am still confused”, and only 5% selecting “I was not confused”. On the topic of participants who wanted to cease the dialogue without the robot’s help in the unproductive confusion condition, 110 out of 140 feedback responses reported “extremely” and “very” levels of abandonment.

Turning to the PARH evaluation, the foundation of our data analysis is based on the assumption that users are unaware of the objective behind our research. The average showed that the average user did not have a clear understanding of the study (*M* = 3.86, SD = 0.72). Following the methodology suggested by the author of the PARH survey Rubin (2016), we investigated the potential significance of the average PARH survey question against a theoretical mean of 4 (*i.e.*, the survey midpoint value). Specifically, a sample t-test shows that the sample mean was significantly less than the score midpoint of 4, therefore participants were generally unclear about the nature of the study (*M* = 3.86, SD = 0.72, *Mdn* = 4), *t*(80) = −1.8, *p* − *value* < 0.05, *d* = 0.039.

## 5 Discussion

As outlined in the introduction, the key challenge for this work is the elicitation and detection of confusion states in the user. Earlier in the paper we expressed this in terms of an overall hypothesis for our work (see Section 1) as well as a set of evidenced individual hypotheses with respect to the modalities that we investigated. Overall, these various questions can be construed as two broad research questions. First, do participants self-recognize different states of confusion—albeit under the conditions set out in a specifically designed HRI study? And second, are there any detectable manifestations of behavior that participants produce which can be used to estimate different states of confusion? In the following, we make a number of observations on the data to help us draw some answers with respect to these questions.

First, it is clear from the results that the participants are well aware that they are confused. Importantly it can be observed that results for Condition B_A1 and Condition B_A2 were very similar as would be expected, as were the results for Condition A1_beforehelp and Condition A2. Digging into more detail, it was apparent that for the productive confusion experimental group, most of the participants were confident that their confusion could be overcome with the help of the robot. And in the unproductive confusion experimental group, most of the participants wanted to stop the current conversation without any help from the robot when they were confused.

Turning to the hypotheses we noted with respect to physical manifestation and the modality data that was subsequently analyzed, we saw that results seen were broadly in line with our expectations in most cases, though it is notable that the subtleties of some analyses did produce some results which were unexpected. Considering first the face based detection of emotion. Here we saw that as expected positive emotions, or rather non-negative emotions “happy” and “neutral” were the main emotions expressed by people who were in a non-confused state during these interactions, while the emotional expression associated with “sad” was correlated with participants in both the productive confusion and the unproductive confusion condition. Turning to the differences between productive and unproductive confusion conditions, for the productive confusion state, participants displayed more instances of positive and surprised emotions in the productive condition; while for the unproductive confusion state, participants displayed more instances of negative emotion.

Considering the related concepts of eye gaze and head pose, we see that these features are potentially strong indicators of confusion states, but that the observed behaviors for these two features are not aligned with each other, and indeed one of our hypotheses were not met. Specifically, eye gaze results, for the productive confusion experimental group, demonstrate that the range of eye gaze for the participants with the help condition is larger than that of the before-help condition. In the unproductive confusion experimental group, the range of users’ eye-gazing in non-confusion condition is larger than in the unproductive confusion. These results, taken together indicate that stronger confusion states are associated with a lower range in eye gaze variances, which is in disagreement with our initial assumptions and hypothesis.

However, for head pose analysis, we observed that the range of head pose before the robot provided help is larger than that for both after the provision of help and in the non-confusion condition. Similarly, for the unproductive confusion group, the head pose angles for participants during unproductive confusion were greater than for those in non-confusion states. These results indicate a tendency to vary head pose more during confusion states which is in accordance with our expectations.

While the visual indicators of confusion seem strong, the indicators associated with speech were more mixed. In particular, no significant differences were found for participants’ emotion pitch across conditions. We note that one reason that might explain this is that voice pitch is differentiated (Aung and Puts, 2020), depends on their gender, ages, *etc.* Since our analysis does not attempt to account for such demographic variation, this may account for the failure to identify meaningful differences. Despite this, a more focused emotion analysis demonstrated a range of potential indicators. As hypothesized, the positive “happy” emotional category for non-confusion dialogues was greater than for both productive and unproductive confusion dialogues. Surprisingly, perhaps, there were no indications of the “sad” emotion in the participants’ speech under those experimental conditions. Moreover, under the productive confusion conditions, the number of “anger” emotions in the participants’ speech before the robot provided help was greater than with the robot’s help. Also, participants were more neutral after the robot helped them overcome their confusion relative to the period before getting the robot’s help.

Silence duration also turned out to be a useful factor to consider in assessing participants’ potential confusion states. In simple terms, the interaction silence duration for non-confusion dialogues is shorter than for those dialogues with confusion states. This difference, as predicted by our hypothesis, can be interpreted, perhaps, as participants taking more time to consider the problem presented to them. However, it should be noted that in the specific case of productive confusion conditions, the difference in interactive silence periods was less pronounced.

A final important comment is that we did not indicate to participants that this study was specifically about confusion or any particular investigation of mental states. Between our understanding of the introductions given and the post-study interviews conducted, we can have some certainty that the participants were not aware of our research purpose. Therefore, we have some confidence that the participants’ behaviors across different confusion and non-confusion conditions are natural—albeit within the context of a semi-controlled HRI study.

## 6 Limitation

While the current investigation has shown strong potential for a systematic detection and later mitigation of confusion effects in interaction, there are of course a number of notable limitations in our experimental data collection and analysis, as presented here. In the following, we briefly outline some of these limitations along with our thoughts on mitigation of these limitations.

The first limitation of note concerns the limited demographic range of our participants. While this study did attempt to target a relatively large pool of participants, the social and cultural background of these individuals is not representative of the general population. We note that 61 out of 81 participants were students in colleges, and of those 61 participants, most of them have a technical background in science and engineering. Moreover, 69 of the 81 participants were between the ages of 18 and 44. Although we opened recruitment to a wider society, we fully acknowledge that those who participated in the study have a higher propensity towards technology and interest in attending this HRI experiment (Henrich et al., 2010; Fischer, 2021).

The second issue of note is that there were 36 participants who did not self-identify as being native English speakers. Among those participants we noted a small trend towards higher confusion reports—which may be in part due to either misunderstandings of the Pepper itself or indeed a lesser ability to understand the problems as articulated. We encountered a similar trend in our earlier pilot study. To mitigate this issue, we designed the “free-talk session” segment of the interaction in order to allow the participants to become comfortable with the mode of communication. We decided not to omit these participants from the study as nonnative speakers are an important component of many large societies.

In our analysis, we have attempted to normalize certain measurements by time to account for the fact that some dialogues which have confusion conditions introduced will often by nature take longer than those without and hence will have higher counts of emotional expression, *etc.* This analysis, however, does not account for the fact that some users in the confusion state often expressed a desire to end a dialogue early and move on. This not only impacts the analysis mechanism, but also means that fewer instances associated with a confused condition could be collected. An alternative strategy would have been to make the participant wait until a minimum time for the dialogue, but we decided that this would add an additional contradiction with boredom, which we were aiming to minimize.

Finally, it is notable that while our analysis did show a strong association between confusion experimental conditions and self-reported confusion states, this is often not fine-grained enough to account for the change in confusion states for a participant over the duration of a given dialogue. Therefore, we believe that a more fine-grained analysis of our data to focus on the specific points at which a third party annotator believes the user has moved to a confusion state could be highly beneficial. Furthermore, in this study, four patterns of confusion stimuli are referred to design interactive dialogues only, and the feature analysis with different confusion states from specific type of confusion stimuli is out of this study scope.

## 7 Future work

While the analysis presented here has attempted to concretely demonstrate the relationships between observable features, confusion conditions, and self-reported confusion states, this is not in itself an operationalizable model, and indeed, there are many other aspects to the data which are beneficial for analysis. Thus, our immediate future work focuses on these joint objectives of extending the analysis of the data and building an operationalizable model as part of a more complete HRI investigation of confusion in interaction.

Specifically, we plan to expand our analysis to include semantic textual features, full-body gestures and stances, and also to assess the impact of emotion arousal and valence on acoustic features in user speech, as documented in previous studies (*e.g.*, Weninger et al. (2013)). This analysis will encompass different confusion states and non-confusion states, or/and a time-domain examination of emotion valence (seen in Deshpande et al. (2019)). As part of this extension, we also plan to provide a more fine-grained though subjective analysis of participant confusion by designing an annotation schema of confusion and non-confusion states for annotators to label those multimodal data. In parallel to this we wish to further delve into the time series nature of the data to uncover the progression of confusion over the course of an interaction and look to see if there are correlations across features as the participant transitions through mental states. Normalizing across participants for differences in duration, *etc.*, will be an important consideration in this work.

While our interest here has been to systematically analyze the features and indicators of confusion, the core outcome from a HRI perspective is to build a machine learning driven confusion estimator model which is incorporated into a real-time conversational process. To that end, we also aim to build a multimodal fusion based model to estimate the confusion score for a given user. While the construction of such a model building on various foundational models and deep learning methods is very feasible, the challenge will be to make such a model generalized in the sense that it can be applied to other experimental scenarios.

Finally, it should be noted that an important part of our ongoing work is the development of conversational policies which can benefit from the identification of users who have entered a confused state (Li and Ross, 2022). In this work, we have developed several operational dynamic dialogue planning policies with specific implementable solutions. The ultimate goal with this work is to design a dialogue framework that integrates these dialogue policies for confusion mitigation, which can be applied to eliminate different states of confusion in the different interacting dialogue systems. While current advancements in large-language model conversational technology do change the overall trajectory of conversational HRI, accounting for personalized assessment of the user during interactions will require much more systematic study and modeling.

## 8 Conclusion

This paper presented a controlled HRI study to investigate the systematic triggering and detection of confusion states in task-oriented interactions. Analysis showed that participants were aware of being confused across the experimental conditions associated with confusion. Furthermore, both visual and speech based signals were shown to have significant correlations with the confusion conditions associated with individual dialogues.

Unlike previous work that focused on a single confusion state with a small number of participants and multiple task types, this paper provided a more systematic investigation across two confusion types and had greater control with respect to the presentation and sequencing of stimuli. We argue that this study validates the earlier study and opens possibilities for including confusion and related task-oriented mental states into a generalized framework for social and affective HRI.

In future work, we plan to extend the analysis of the data introduced in this paper, generate abstracted confusion assessment models that should be generalizable to other interactive systems, and build out a dialogue framework that includes policy elements that are sensitive to confusion and subsequently assist users to overcome confusion states before disengagement results. Taken together, we believe that this is a small but important step towards true social intelligence in HRI.

## Data Availability

The datasets presented in this study can be found in online repositories. The names of the repository/repositories and accession number(s) can be found below: https://github.com/nalibjchn/SituatedHRITrackConfusion/tree/main/SharedFeatureData.

## Footnotes

1
https://github.com/nalibjchn/SituatedHRITrackConfusion

2
https://github.com/nalibjchn/SituatedHRITrackConfusion/blob/main/DialogueScripts.pdf

3
https://pypi.org/project/fer

4 More details on the post-study survey are available here: https://github.com/nalibjchn/SituatedHRITrackConfusion/blob/main/Surveys.pdf
